# Editorial: Non-coding RNA regulation of secondary metabolism in plants

**DOI:** 10.3389/fpls.2024.1382709

**Published:** 2024-03-11

**Authors:** Caini Wang, Shaohua Zeng, Yongliang Liu, Jiabao Ye, Feng Xu

**Affiliations:** ^1^ College of Horticulture and Gardening, Yangtze University, Jingzhou, China; ^2^ South China Botanical Garden, Chinese Academy of Sciences (CAS), Guangzhou, China; ^3^ Department of Plant and Soil Sciences and Kentucky Tobacco Research and Development Center, University of Kentucky, Lexington, KY, United States

**Keywords:** secondary metabolism, lncRNA, miRNA, regulatory mechanism, plant

Secondary metabolites have been a recent Research Topic for many years in plants. What we usually see, smell and taste are the primary metabolites that give plants their color, scent and acidity. However, the synthesis and regulation of secondary metabolites, such as terpenoids, flavonoids, alkaloids, phenolic acids, and other secondary metabolites, directly affect the production of primary metabolites in plants. They are multifunctional, defending against natural enemies, resisting insects and diseases, signaling, and producing medicinal components that are beneficial to the human body, among other things. Based on this, the biosynthesis and regulation of plant secondary metabolism has been highly active Research Topic for many years. Many researchers have been studying the complex regulatory network formed by enzyme genes and transcription factors (TFs) to understand the synthesis and regulation of these secondary metabolites. In recent years, more and more studies have revealed that non-coding RNAs are involved in this regulatory network, mediating the expression of TFs indirectly affecting the biosynthesis of plant secondary metabolites.

Non-coding RNAs are a class of RNAs that do not code for proteins, including short-stranded miRNAs and long-stranded is concerns. They can attach to multiple target genes or multiple non-coding RNAs that interact with the same protein, DNA, or RNA, and thus play a role in cellular functions. In this Research Topic, we present many articles that have provided new insights into plant secondary metabolism biosynthesis. Lin et al. compared the red leaves of the bud mutation branches of *Acer pictum subsp. mono* with those of the green leaves of the wild-type branches and found a significant decrease in chlorophyll and carotenoids, and a significant increase in anthocyanins and various antioxidant enzymes in red leaves. The miR160b targeting *ApSUS*, miR6300 targeting *ApTPR*, *ApUFGT*, and *ApNRT*, and miR396g targeting *ApUGP2* were detected by transcriptome and miRNA sequencing, which suggests that miR160b, miR6300, and miR396g are the key miRNAs for the regulation of the stabilization of anthocyanin accumulation in the process of leaf color change. Hou et al. revealed that 6 mM exogenous salicylic acid (SA) induced the accumulation of folate (5-methyltetrahydrofolate) and methionine (Met) in *Setaria italica* (L.) P. Beauv. Hou et al. found that in *S. italica*, the expression level of the *DHFR1* gene, which is associated with folate biosynthesis, was up-regulated in Nov-m0139-3p. On the other hand, *DHFR2* was down-regulated in Nov-m0731-5p and up-regulated 4.27-fold in Nov-m0461-5p and 1.32-fold in Nov-m0664-3p. Additionally, CYSC1, which is associated with Met synthesis, was regulated by Nov-m0319-3p, Nov-m0416-5p, and Nov-m0042-5p.

Along with this, the researchers found that non-coding RNAs can be modified by N^6^-methyladenosine (m^6^A) and these RNAs can have important functions during plant growth. Rudy et al. revealed that m^6^A can post-transcriptionally modify RNA and induce leaf senescence in barley. Additionally, transgene is particularly susceptible to small RNA (sRNA)-mediated silencing, and different terminators can differentially affect the production of siRNAs at the level of the transgene and its expression. de Felippes et al. identified the HSP18.2 terminator in *Arabidopsis thaliana* that improves high-level transgene expression, thus providing a new strategy for achieving robust and efficient transgene expression.

Besides miRNA gene expression, which is involved in plant growth and development and secondary metabolism, lncRNAs can also be involved in the regulation of plant growth and development through transcriptional and post-transcriptional regulation of gene expression. Jiang et al. found an increase in seed germination and a significant decrease in seed dormancy during seed maturation by treating wheat seeds at high and ambient temperatures. Whole-transcriptome sequencing analysis revealed that the high-temperature-mediated gibberellin (GA) biosynthesis gene *TaGA20ox1* (TraesCS3D02G393900) was involved in dormancy, and the *TaCDPK21* (TraesCS7A02G267000) gene of the calcium signaling pathway showed a negative regulatory role in dormancy. In addition, not only the newly identified miR27319, which is located at the key node of this regulatory network, acts positively in seed dormancy, but also *TaDOG1-3A, GID1-1D*, *TaMFT-3A* and *TaMFT-3D* target novel lncRNAs (MSTRG.4103020.1, MSTRG.861889.1, MSTRG.729470.1 and MSTRG.891662.2) to regulate the molecular mechanism of seed dormancy.

Here, we report that noncoding RNAs are involved in the biosynthesis and regulation of plant secondary metabolites, providing valuable insights into the study of natural plant secondary metabolism. So far, the regulation mechanism of secondary metabolite biosynthesis has attracted extensive attention, and many of studies have focused on the key genes in the secondary metabolite biosynthesis pathway. In the future, it is crucial to invest in the functional studies of non-coding RNAs related to secondary metabolite biosynthesis, which is conducive to the in-depth unraveling of complex secondary metabolite biosynthesis mechanisms and metabolic pathways.

## Author contributions

CW: Writing – original draft. SZ: Writing – review & editing. YL: Writing – review & editing. JY: Writing – original draft, Writing – review & editing. FX: Writing – original draft, Writing – review & editing.

